# The long-term persistence of the *w*Mel strain in Rio de Janeiro is threatened by poor integrated vector management and bacterium fitness cost on *Aedes aegypti*

**DOI:** 10.1371/journal.pntd.0013372

**Published:** 2025-07-23

**Authors:** Márcio Galvão Pavan, Franck Jeannot Gnonhoue, Jessica Corrêa-Antônio, Karine Pedreira Padilha, Gabriela Azambuja Garcia, Felipe de Oliveira, Luiz Paulo Brito, Luciana Dias, Ademir Jesus Martins, Vincent Corbel, José Bento Pereira Lima, Gabriel Luz Wallau, Ary Hoffmann, Oswaldo Gonçalves Cruz, Daniel Antunes Maciel Villela, Rafael Maciel-de-Freitas

**Affiliations:** 1 Laboratório de Mosquitos Transmissores de Hematozoários, Instituto Oswaldo Cruz, Rio de Janeiro, Brazil; 2 Laboratório de Biologia de Tripanosomatídeos, Instituto Oswaldo Cruz - IOC, Rio de Janeiro, RJ, Brazil; 3 Laboratório de Biologia, Controle e Vigilância de Insetos Vetores, Instituto Oswaldo Cruz, Rio de Janeiro, Brazil; 4 Instituto Nacional de Ciência e Tecnologia em Entomologia Médica, Universidade Federal do Rio de Janeiro, Rio de Janeiro, Brazil; 5 MIVEGEC, IRD, CNRS, University of Montpellier, Montpellier, France; 6 Departamento de Entomologia e Bioinformática, Instituto Aggeu Magalhães, Fiocruz Pernambuco, Recife, Brazil; 7 Department of Entomology and Arbovirology, Bernhard Nocht Institute for Tropical Medicine, Hamburg, Germany; 8 Universidade Federal de Santa Maria, Rio Grande do Sul, Brazil; 9 Pest and Environmental Adaptation Research Group, School of BioSciences, Bio21 Institute, The University of Melbourne, Melbourne, Australia; 10 Programa de Computação Científica, Rio de Janeiro, Brazil; Instituto Leonidas e Maria Deane / Fundacao Oswaldo Cruz, BRAZIL

## Abstract

New tools and methods are currently under evaluation by the World Health Organization for preventing arbovirus transmission, such as dengue, Zika, and chikungunya. One promising approach involves deploying *Aedes aegypti* with the endosymbiotic bacterium *Wolbachia pipientis* to disrupt arbovirus transmission within endemic urban environments. The release program of mosquitoes with the *Wolbachia*’s *w*Mel strain started in August 2017 in 6.88% of the city area of Rio de Janeiro, where 13.1% of the city’s population live (~890,000 inhabitants). The deployment of *Wolbachia w*Mel strain in Rio finished in December 2019 with a suboptimal 32% introgression of *w*Mel strain, which coincided with a 38% and 10% reduction of dengue and chikungunya, respectively. We conducted an independent evaluation during 20 consecutive months to evaluate whether the *w*Mel distribution and frequency would expand or retract. More than 50,000 mosquitoes were sampled in 12 neighborhoods with estimated 500,000 inhabitants, of which 39.2% were *Ae. aegypti*. In total, 7,613 of 19,427 collected *Ae. aegypti* were screened individually for *w*Mel. Climate, environmental and insecticide application data was used to model the spatiotemporal introgression of *w*Mel. The routine insecticide rotation adopted by the Brazilian Ministry of Health caused the crash of both *w*Mel-infected and -uninfected populations shortly after an increase in coverage with spinosad. However, the *w*Mel-uninfected mosquitoes recovered soon to levels even higher than before, whereas the *w*Mel-infected failed to recover after the population crash. The well documented fitness cost of *w*Mel in egg hatching leads to the absence of an egg bank necessary to recover after adult population was disrupted. Finally, we observed the mtDNA haplotype associated with released *Wolbachia* at a frequency of ~25% in field-caught uninfected mosquitoes. The reason underlying the poor introgression of *Wolbachia w*Mel strain is multifold. The adoption of an effective larvicide that crashed both *w*Mel-infected and -uninfected populations, the absence of an egg bank due to high fitness cost of egg hatching in the *w*Mel-infected mosquitoes, a suboptimal *Wolbachia* invasion before the intervention, and *Wolbachia* loss synergically contributed to the lower invasion and, by corollary, modest epidemiological outcome in Rio de Janeiro. Our results highlight the need to plan and implement technical guidance on Integrated Vector Management in Brazil prior and during the nationwide release of *Wolbachia*-infected mosquitoes to optimize dengue mitigation efforts while ensuring the judicious use of resources.

## Author summary

In Rio de Janeiro, efforts to control diseases like dengue and chikungunya involve a myriad of approaches, including targeting breeding sites, insecticide application, engaging communities on good practices, and the deployment of *Wolbachia w*Mel strain. By the end of 2019, only about a third of the field-caught *Aedes aegypti* population carried *Wolbachia* and coincided with a 38% drop in dengue cases and a 10% reduction in chikungunya—good, but not as effective as hoped. To see the long-term stability of the *w*Mel strain in Rio de Janeiro, we independently collected over 50,000 mosquitoes from neighborhoods home to around half a million people and analyzed nearly 40% of the *Aedes aegypti* for *Wolbachia*. Biotic and abiotic variables such as temperature, other mosquito species, vegetation coverage, and the use of insecticides were estimated to model the *w*Mel introgression. The substitution of the analogue of juvenile hormone (pyriproxyfen) by the bioinsecticide spinosad wiped out most mosquitoes—both those with and without *Wolbachia*. However, mosquitoes without *Wolbachia* bounced back quickly, even growing in high number. Meanwhile, the *Wolbachia*-infected mosquitoes struggled to recover. This was likely because they have a disadvantage when egg-hatching compared to native mosquitoes, meaning *w*Mel-infected females couldn’t “store” enough eggs to rebuild their population. On top of this, some mosquitoes lost their *Wolbachia* infection over time, making it even harder for the bacteria to spread. Together, these factors explain why *Wolbachia* mosquitoes didn’t recover as expected, limiting their impact on dengue transmission. Therefore, to make these mosquito-based programs work better in Brazil, planning their deployment must involve delving deeper into the issue of integrated vector management to make sure resources are used wisely.

## Introduction

Arboviruses such as dengue (DENV), Zika (ZIKV), and chikungunya (CHIKV) are a global public health concern, particularly in tropical and subtropical regions. With an estimated 3.9 billion people at risk of infection, and approximately 390 million infections annually, dengue imposes a substantial burden on healthcare systems and communities worldwide [1,2]. CHIKV entered the spotlight of global health concerns in the early 2000s, following the emergence of a new epidemic strain originating from an enzootic lineage. In 2014, Brazil experienced its first autochthonous cases of CHIKV, attributed to both the Asian and the East Central South Africa (ECSA) genotypes of the virus, with the latter rapidly disseminated throughout the country [3]. Around the same time, the world bore witness to the swift spread of ZIKV across the Western Hemisphere within the span of a year. ZIKV made its debut in Brazil in 2014 after being introduced from the Pacific Islands. Just a year later, a surge in cases of microcephaly among newborns prompted the World Health Organization (WHO) to declare a Public Health Emergency of International Concern [4,5].

The above-mentioned arboviruses are primarily transmitted by the mosquito *Aedes aegypti*, emphasizing the pivotal role of targeting mosquito population to mitigate disease transmission considering that pharmacological interventions are still limited for these neglected viruses. This anthropophilic species is found mostly in urbanized areas with low vegetation coverage, and is closely associated with humans for several reasons, with females preferring to feed on human blood, laying eggs in artificial breeding sites near human dwellings and having a short flight range around hosts and breeding sites [6–9]. Current vector control programs in Brazil primarily rely on insecticide use and campaigns targeting immature breeding sites to curb vector abundance. However, the widespread prevalence of insecticide resistance in native *Ae. aegypti* populations coupled with logistical challenges in implementing larval source reduction measures across sprawling urban areas limits the effectiveness of traditional control methods in this context [10]. Therefore, the development of innovative strategies to complement existing vector control efforts is a priority for managing mosquito-borne diseases.

New tools and methods are currently under evaluation by the WHO-VCAG for dengue prevention, such as genetic manipulation, spatial repellents, vector traps, housing modification and pathogen transmission reduction through microorganism (*cf.* https://www.who.int/groups/vector-control-advisory-group/summary-of-new-interventions-for-vector-control). Among them, one promising approach involves deploying the endosymbiotic bacterium, *Wolbachia pipientis*, to disrupt arbovirus transmission within endemic urban environments [11]. While naturally absent in *Ae. aegypti*, *Wolbachia* strains have been successfully transinfected into *Ae. aegypti* from other insect species, some with potent pathogen-blocking properties [12–14]. *Wolbachia* strains can spread through arthropod populations though a combination of efficient maternal transmission, which depends on high *Wolbachia* density in female reproductive systems, and their ability to cause cytoplasmic incompatibility (CI), where uninfected females are at a disadvantage over infected females because they fail to produce offspring when mated with infected males [15]. It is thereby possible to replace local *Ae. aegypti* populations in disease-endemic areas that are highly susceptible to arboviruses with *Wolbachia*-infected equivalents that have a reduced potential for pathogen transmission. Another application of *Wolbachia* in arbovirus mitigation involves mosquito population suppression by releasing only *Wolbachia*-infected males; when wildtype females mate with these males, they only lay non-viable eggs due to CI [16].

Successful releases of *Wolbachia* using both *w*Mel and *w*AlbB strains, replacement or suppression strategies, have been carried out in countries such as Australia, Brazil, Colombia, Indonesia, Malaysia, Singapore, and Vietnam [16–21]. Data from releases across increasingly larger areas have been published, demonstrating *Wolbachia*’s capability to introgress into native *Ae. aegypti* populations. Additionally, reports showcasing the epidemiological impact of *w*Mel and *w*AlbB on reducing dengue transmission in larger urban settings are emerging [22].

The inaugural release of *Wolbachia* in the Americas occurred in the secluded village of Tubiacanga, Rio de Janeiro, Brazil. After 20 weeks of consecutive releases, however, the frequency of *w*Mel decreased significantly due to the widespread use of pyrethroids by local households for personal protection against mosquito bites [18]. This observation highlighted the critical importance of matching the genetic compatibility between the released strain and the native population to ensure successful *Wolbachia* invasion [23]. Subsequently, *w*Mel introgression was achieved through a second round of releases of mosquitoes backcrossed with field mosquitoes of Rio de Janeiro naturally resistant to pyrethroids [18].

After overcoming initial challenges, *Wolbachia* deployment in Rio de Janeiro was expanded and also took place in the neighboring city of Niterói. In Niterói, *w*Mel mosquitoes were deployed to the full extension of the city limits, which the authors reported to be associated with substantial reductions in dengue, chikungunya, and Zika incidence rates compared to predefined control areas [24]. However, outcomes in Rio de Janeiro were more modest. Despite releasing 67 million *w*Mel *Ae. aegypti* mosquitoes between 2017 and 2019, an average of only 32% *w*Mel introgression level into the wild population was achieved, which the authors reported to be associated with a reduction of 38% and 10% in dengue and chikungunya notifications, respectively [25]. While the results in Rio de Janeiro remain potentially encouraging, the barriers hindering *Wolbachia* introgression and epidemiological impacts warrant further investigation.

From 2020 to early 2023, the World Mosquito Program (WMP) suspended *w*Mel deployment in Rio de Janeiro, which had covered approximately 6.88% of the city’s area. Considering mosquito biology and the effects of *Wolbachia* on invertebrate hosts, we were interested in testing whether the distribution of *w*Mel would expand or contract over time without additional releases. To address this, we present herein an independent dataset from WMP comprising extensive mosquito sampling over 18 months within the boundaries of the *w*Mel released area in Rio de Janeiro. Furthermore, we explore possible factors underlying the partial invasion of *w*Mel in Rio and an unexpected contraction of its geographic distribution and prevalence we have documented within the city.

## Methods

**Fig 1:**
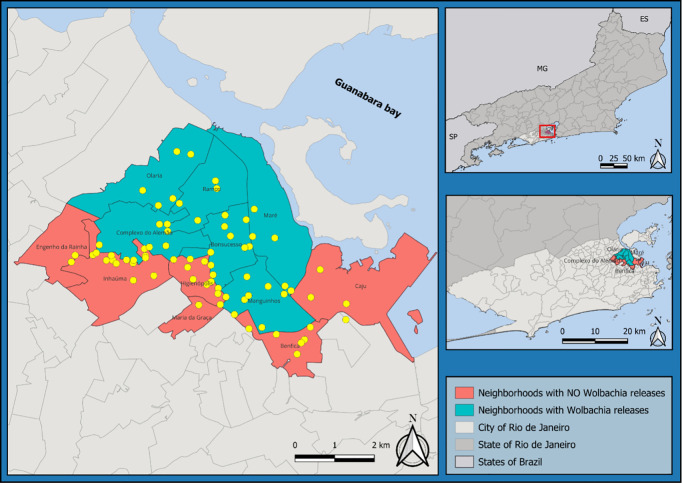
Map of Rio de Janeiro showing the study area. BG-sentinel traps are shown in yellow. The base layer map was downloaded from the open-source site of IBGE: https://www.ibge.gov.br/geociencias/downloads-geociencias.html.

**Study area.** Rio de Janeiro is the second largest city in Brazil, with 6.75 million inhabitants over an urban area of 1260 km^2^. According to the Köppen climate classification, Rio de Janeiro exhibits a combination of tropical savanna (Aw) and tropical monsoon (Am) climates, marked by extended periods of intense rainfall, particularly from December to March [26]. The city experiences hot and humid conditions during the summer, with warm and sunny winters. In the inland regions of the city, where our activities took place, summer temperatures can exceed 40°C, although such extremes are typically short-lived. Maximum temperatures above 27°C are observed monthly (Fig 1).

The *w*Mel release program started in August 2017 and lasted up to December 2019, i.e., a total period of 29 months. No further releases were conducted after this period until the end of this study. Therefore, monitoring the persistence of *Wolbachia* frequency in such areas is critical to assess the success of the large deployment conducted across Rio de Janeiro. The release zone covered a total area of 86.8 km^2^ (6.88% of total city area) with around 890,000 inhabitants representing 13.1% of the city’s population [25]. Our trapping network was placed at the southern limit of the *w*Mel released area in Rio de Janeiro to test whether the *w*Mel distribution expanded to adjacent neighborhoods that have not received *Wolbachia* deployment, or whether it had contracted to a smaller geographic area after releases stopped in December 2019. We covered a total of 12 urban neighborhoods within an area of 33.5 km^2^ (38.6% of the WMP release area) where approximately 495,000 people live (55.6% of the population under *w*Mel intervention) (Fig 1). This is a dangerous region due to the high number of Brazilian slums (favelas) with armed drug dealers present. These areas are hard to access, with a low-level presence of basic government services and a low Human Development Index (S1 Table).

**Mosquito sampling.** Between August 2021 and March 2023 (20-month timeframe), we installed 75 BG-Sentinel traps in 12 neighborhoods to track changes in the spatiotemporal distribution of *Wolbachia* after releases stopped (Fig 1). Traps were visited fortnightly and captured mosquitoes were identified using local taxonomic keys [27]. A subset of mosquitoes identified as *Ae. aegypti* were individually screened for *Wolbachia*. When 24 or fewer *Ae. aegypti* were collected in a single BG-Sentinel trap, all specimens were screened for *Wolbachia*. If 25–50 were collected, we selected 25 specimens for screening, while if >50 individuals were collected, we screened 30.

***Wolbachia* screening.** Adult mosquitoes were placed individually into 2 mL microtubes with 3.5 mm glass beads and 100 uL of a buffer (10 mM Tris-Cl, 1 mM EDTA, 25 mM NaCl, pH 8.2) with 1uL of Proteinase K (250 ug/mL), and ground into powder in the Tissue Lyser II (Qiagen, Hilden, Germany) for 60s at 50 Hz. Samples were incubated at 56 °C for 30 min, followed by 98 °C for 15 min to interrupt the Proteinase K activity. Multiplex PCR was carried out according to Walker et al. [28] with 10 pmoles of each one of the primers that amplify a fragment of the *wsp* gene of *Wolbachia* (185 base pairs) and of the ribosomal protein S17 (RPS17) of *Ae. aegypti* (305 bp), ~ 10ng of DNA, and 12.5 uL of the GoTaq Green Master Mix (Promega, Madison, USA) in a total 25uL reaction.

***Wolbachia* quantification in field sampled mosquitoes.** Mosquito samples collected in both the first *Wolbachia* release area of Rio de Janeiro (Tubiacanga) and in one of the 12 neighborhoods with regular sampling (Bonsucesso) were used to estimate *Wolbachia* densities in whole mosquito bodies between 2019–2023, i.e., before our field monitoring has started. DNA was extracted with the Nucleospin Tissue kit (Macherey-Nagel, Düren, Germany), following the manufacturer’s protocol. The relative quantification of *Wolbachia* was performed in the QuantStudio 6 Flex Real-Time PCR System (Applied Biosystems, Waltham, MA, USA) with TaqMan Fast 1-Step Master Mix (Applied Biosystems, Waltham, MA, USA), using specific primers that amplify a fragment of the WD0513 gene, and a fragment of the *Ae. aegypti* ribosomal protein S17 as internal control, as previously published [29].

**Mitochondrial lineage detection**. Australian *Ae. aegypti* mosquitoes infected with *Wolbachia* had been backcrossed with the local mosquito population to acquire a Brazilian genetic background prior to releases in Rio de Janeiro [18]. Due to the maternal inheritance of *Wolbachia*, the mitochondrial lineage of all *w*Mel-infected *Ae. aegypti* is from the Australian mosquitoes [18,30], and thus it is possible to detect the loss of the bacteria in field mosquitoes (i.e., negative for *Wolbachia* detection, but mitochondrial haplotype of Australia). Considering that *w*Mel is adversely affected by heat stress [31,32], we selected *w*Mel negative samples from all localities collected in January/February 2023, when the highest summer temperatures are usually recorded in the city, and sequenced a 866-bp fragment of the mitochondrial cytochrome oxidase I. Briefly, we reextracted the DNA of samples with the DNA Nucleospin Tissue kit (Macherey-Nagel, Düren, Germany), following the manufacture’s recommendations and the amplicons were produced through PCR [33]. The PCR products were purified with the GFX PCR DNA and Gel Band Purification kit (Cytivia, Marlborough, MA, United States) and both DNA strands were subjected to Sanger sequencing reactions with the ABI Prism BigDye Terminator v3.1 Cycle Sequencing kit (Thermo Fisher Scientific, Walthan, USA) and run on an ABI 3730 automated sequencer (Applied Biosystems, Foster City, USA). We removed primer sequences and edited out both forward and reverse strands for each sample using SeqMan Lasergene v.7.0 (DNAStar, Inc., Madison, Wisconsin, USA). The genealogy of DNA sequences was inferred with NETWORK 10.2 (Fluxus-Engineering), using the Median-Joining network method, with a Maximum-Parsimony (MP) calculation in the post-processing. We added in the analysis representative sequences retrieved from GenBank (https://www.ncbi.nlm.nih.gov/genbank/) from samples collected in South America, including Brazil (GenBank accession KU936162, JX456411, KM203142, KM203172), and in Australia (GQ143718, OM214532).

**Capture data pre-processing.** The number of mosquitoes captured per trap was considered for all areas. Mosquitoes were grouped by neighborhood of collection and by sets of neighborhoods that had *Wolbachia* mosquito releases and those that did not. The proportion of *Wolbachia*-infected mosquitoes was estimated as the frequency of mosquitoes with *Wolbachia* among the number of tested mosquitoes. Environmental variables (temperature and precipitation) were normalized by differences from mean values and divided by the standard deviations.

**Climate and Environmental Data Acquisition.** The cloud-based geospatial data processing and analysis platform of Google Earth Engine (GEE) (https://developers.google.com/earth-engine/datasets) [34] was used to acquire climatic and other environmental variables. Land surface temperature information was obtained from the MOD11A1V6.1 dataset (https://developers.google.com/earth-engine/datasets/catalog/MODIS_061_MOD11A1) band (LST_Day_1km) with a spatial resolution of 1 km and a temporal resolution of 8 days [35]. Landsat 9 Level 2, Collection 2, Tier1 (https://developers.google.com/earthengine/datasets/catalog/LANDSAT_LC09_C02_T1_L2) provided “Band 10 surface temperature” with a spatial resolution of 30 meters and a temporal resolution of 16 days [36]. A landscape variable was obtained through the Normalized Difference Vegetation Index (NDVI) generated from images of the COPERNICUS/S2 dataset (https://developers.google.com/earthengine/datasets/catalog/COPERNICUS_S2_HARMONIZED) Band“4 (Red) and Band 8 (NIR) dividing NIR-Red by NIR + Red, with a spatial resolution of 10 meters and a temporal resolution of 5 days [37]. Images were retrieved using the shapefile of Rio de Janeiro delineating the study areas and imported into the GEE platform, selecting the period of the study (from August 2021 to March 2023) and images with up to 10% cloud cover. Daily meteorological data time series including maximum and minimum temperatures from August 2021 to March 2023 were obtained from the automatic station A652 from the meteorological database of the Brazilian National Institute of Meteorology (INMET) (https://portal.inmet.gov.br/). For each trap verification day, the average of maximum and minimum temperatures and precipitation were calculated. Climate and environmental data were extracted from GEE images with QGIS Quantum GIS v. 3.22 [38]. Geographic coordinates of trap locations were imported, and climate and environmental data were obtained according to trap location and verification date using the Point Sampling Tool plugin. Finally, a CSV file was generated containing GEE data grouped with meteorological data from INMET.

**Spatial analysis.** We investigated the *w*Mel strain spatial distribution in Rio de Janeiro during the 20 months of observations. Given the changes in *Wolbachia* frequency during our study period (see below), we divided our time series into four periods of five months each: (A) Aug.2021-Dec.2021, (B) Jan.2022-Mai.2022, (C) Jun.2022-Oct.2022, and (D) Nov.2022-March.2023. A Generalized Additive Model (GAM) was fitted to the proportions of mosquitoes positive for *Wolbachia* by each of the four study periods to observe the spatial dynamics of *w*Mel. The model used a binomial family and the number of captured mosquitoes as weights, with the fit being done by restricted maximum likelihood (REML) [39]. Spatial smoothing was obtained using a 2D spline of the trap coordinates and whether the trap was in an area with previous *w*Mel release as a co-variate. The analysis was done with the MGCV package [40].

**Insecticide resistance: pyrethroids and Spinosad.** The desirability of matching genetics of released and native *Ae. aegypti* populations to achieve *Wolbachia* introgression was well demonstrated in Rio de Janeiro, with particular interest to equivalent insecticide resistance profile. Therefore, we evaluated the insecticide resistance to the larvicide in use by local Government (spinosad), and the most commonly used adulticide (pyrethroids). Routine entomological surveillance involves local health agents seeking potential mosquito breeding sites. After oral consent from householders, breeding sites are removed or destroyed whenever possible, with permanent water-storage containers treated with larvicide following WHO recommendations. The timeframe of this study captured a switch of larvicide in Rio de Janeiro: in December 2021, the analogue of juvenile hormone (pyriproxyfen) was replaced by the bioinsecticide spinosad, with applications continuing until the end of this study.

We assessed *Ae. aegypti* susceptibility to the larvicide spinosad (Natular 20EC– 20.6% spinosad a.i., Clarke Mosquito Control Products, Inc., St. Charles, USA) in laboratory conditions with third-instar larvae of both the *Wolbachia*-infected and -uninfected populations. The *Wolbachia*-infected population was the F2 generation of mosquitoes sampled in the neighborhood of Tubiacanga, the first release site and with a stable *Wolbachia* infection (>95%) [18]. The *Wolbachia*-uninfected population was a field population from the neighborhood of Urca, a site ~20 km distant from release zone. The Rockefeller strain was used as an internal quality control and as a reference lineage for susceptibility [41]. All groups were exposed to 11 spinosad concentrations, ranging from 60 to 600μg/L. Four replicates were conducted for each concentration, with 20 third-instar larvae used in each replicate. The larvae were placed in transparent plastic cups containing 100mL of a spinosad solution prepared using ethanol and dechlorinated water. The control group was exposed to ethanol (0.4%) only. Mortality was registered 24 hours after exposure. The experiments were repeated three times on separate days. To determine the lethal concentrations (LCs), resistance ratios (RR), and their corresponding confidence intervals, the BioRssay software package in the R environment was used for analysis [42]. Resistance ratios (RR_95_) were calculated by dividing the LC_95_ of the tested populations by the corresponding LC of the Rockefeller strain. Linear regression was employed to assess the level of heterogeneity among populations using GraphPad Prism v. 5.0 (GraphPad Software, San Diego, USA).

The frequency of knockdown resistance (kdr) alleles in the voltage-gated sodium channel (Na_V_) was estimated from mosquitoes collected in January/February 2023 as a key genetic marker of pyrethroid resistance. In *Ae. aegypti* populations from Rio de Janeiro, two kdr alleles are known to play a significant role as key mechanisms of resistance, defined by non-synonymous substitutions at the 1016 and 1534 Na_V_ sites of the susceptible genotype Na_V_S (1016 Val^+^ + 1534 Phe^+^): Na_V_R1 (1016 Val^+^ + 1534 Cys^kdr^), with a moderate fitness cost to mosquitoes, and Na_V_R2 (1016 Ile^kdr^ + 1534 Cys^kdr^), with a severe fitness cost [43,44]. A customized Taqman genotyping assay with wild-type and mutant specific probes was performed for the two polymorphic sites separately [45], using for each mosquito sample 5µL of TaqMan Genotyping Master Mix (Thermo Fisher Scientific, Waltham, USA), 0.5 µL of 20X probes and 20ng of DNA, and run in the QuantStudio 6 Real-Time Thermal Cycler (Thermo Fisher Scientific, Waltham, USA).

**Analysis of *Wolbachia* frequency dynamics.** The dynamics of *Wolbachia* frequency is expected to depend on key biological differences between *Wolbachia*-infected and *Wolbachia*-uninfected mosquitoes, such as adult recruitment rates, mortality, vertical transmission, and cytoplasmic incompatibility. We applied a stochastic differential equation model that described the frequency of *Wolbachia*. The model contains a system of equations with variables as the proportion of *Wolbachia* mosquitoes (V), and the total number of captured mosquitoes (S). The model departs from Keelling et al. [46] with various changes in our study. First, the model was modified to have stochastic effects. The frequencies of variables are transformed on a logit scale and mosquito counts on logarithmic scale to facilitate numerical analysis. In the deterministic version of this model, the dynamics of these variables are described by a system of two differential equations:


$$\frac{1}{V(1-V)}\frac{dV}{dt}=b_{i}\left(\tau -(1-\tau )\frac{V}{1-V}(1-qV)\right)-\left(d_{i}-d_{u}\right)S$$

$$\frac{1}{S}\frac{dS}{dt}\;=\;b_{i}\;V\;(\tau -(1-\tau )(1-qV))\;+\;b_{u}\;(1-V)(1-qV)\;-\;(d_{i}\;V\;+\;d_{u}\;\left(1-V)\right),$$

where q describes the proportion of success of cytoplasmic incompatibility, τ is the proportion of vertical transmission, $b_{i}$ is the adult recruitment rate for *Wolbachia*-infected mosquitoes, $b_{u}$ is the adult recruitment rate for *Wolbachia*-uninfected mosquitoes and *d*_*i*_ and *d*_*u*_ are, respectively, mortality rates for *Wolbachia*-infected and uninfected mosquitoes. These adult recruitment rates were evaluated by a basic adult recruitment rate and possible effects due to covariates. This construction follows multiplicative effects in the following way:

${log(b}_{i}=\;log(b_{i,0})\;+\;\Sigma \;\beta_{i,j}\;X_{j}$ and ${log(b}_{u}=\;log(b_{u,0})\;+\;\Sigma \;\beta_{u,j}X_{j}\;,$

where $b_{i,0}$ and $b_{u,0}$ are the basic adult recruitment rate for *Wolbachia*-infected and *Wolbachia*-uninfected mosquitoes, respectively, $X_{j}$, 1 < j < 7, are covariates given by insecticide treatment, NDVI, air temperature, land temperature, presence of other mosquito species, rainfall, insecticide treatment intensity and change from pyriproxyfen to spinosad, and finally $\beta_{i,j}$ and $\beta_{u,j}$ which are the coefficients associated with these variables. NDVI, air temperature, and land temperature estimations were obtained as previously described. The presence of other mosquito species was evaluated through logarithmic numbers of *Culex* sp. and *Ae. albopictus* mosquitoes caught in the traps. The covariate of rainfall was given by the logarithm of rolling sum of rainfall quantities over 28 days, additionally lagged by 14 days. The insecticide treatment intensity was given by the number of households receiving insecticide application, which was counted weekly. Missing data were treated by averaging previous and next observations. The larvicide regime change was coded as 0 before application of spinosad regime in Jan 1, 2022, plus a lag period to be selected from 1 to 6 months.

The analytical approach is to derive a system of stochastic differential equations modifying the deterministic system adding components for drift and diffusion. The diffusion components are given by

$\sigma_{V}=\;b_{i}\;\left(\tau -(1-\mathrm{\backslash taufrac}V1-V(1-qV)\right)+\left(d_{i}-d_{u}\right)S$ for variable V and

${\sigma_{S}\;=\;b}_{i\;}V\;(\tau \;-\;(1-\tau )(1-qV)+\;b_{u}(1-V)\;(1-qV)\;+\;(d_{i\;}V\;+\;d_{u\;}(1-V))$ for variable S. The drift components are given by the right terms in the deterministic system.

This framework with a proper change of variables enables to apply a Euler-Maruyama scheme with a Brownian process, weighting by the variance given by the sum of effects. The change of variables is given by x = logit(V) and y = log(S). Finally, the Euler-Maruyama scheme is applied as

$x_{t\;}\;=\;x_{t-1\;}+\;\delta \;\varepsilon \;+\;\sqrt{\sigma_{V\;}\varepsilon }\;W_{1}$ and $y_{t\;}\;=\;y_{t-1}+\;\delta \;\varepsilon \;+\;\sqrt{\sigma_{S\;}\varepsilon }\;W_{2}$, where $W_{1}$ and $W_{2}$ are Brownian processes N(0,1).

This statistical model was used to evaluate the recruiting rate of adult mosquitoes and the possible effects of other external variables. In this case, we selected a subset containing six neighborhoods with the largest number of observations during the study in the capture data, of which three had *Wolbachia* mosquitoes released (Bonsucesso, Complexo do Alemão, and Manguinhos), and three had not (Benfica, Caju, and Higienópolis).

The inference of parameters was done for parameters basic recruitment rate $b_{i,0},\;b_{u,0}$, and covariate effects $\beta_{i,j},\;\beta_{u,j}$. The level of cytoplasmic incompatibility in the model was assumed to be 99% and vertical transmission set as τ=0.98, based on previous studies performed in Brazil that indicated high levels of cytoplasmic incompatibility and vertical transmission [47]. The value of mortality rates (parameters *d*_*i*_ and *d*_*u*_ in the model) for *Wolbachia*-infected and Wolbachia-uninfected mosquitoes was adjusted according to the measurement period using a daily survival probability of 0.8, based on estimates obtained in previous capture recapture studies with *Wolbachia*-uninfected *Aedes* mosquitoes in Rio de Janeiro. Inference was done with MCMC simulations with the model written for Rstan tool [48]. MCMC was performed using 3 chains with 10,000 iterations each (with 9,000 as burn-in). Different values of lag periods were used for the larvicide change time and a lag of 4 months gave best convergence. These estimations allowed us to obtain intervals of uncertainty. Therefore, it is possible to run a model without external variables and to alternate between the high and low levels estimated for the adult recruitment parameter.

**Role of the funding source.** The sponsor of the study had no role in study design, data collection, data analysis, data interpretation, or writing of the report.

## Results

We collected 50,240 adult mosquitoes (*Culex* sp., *Ae. albopictus* and *Ae. aegypti*) during 20 months at 12 urban neighborhoods of Rio de Janeiro, Brazil, an area of 33.5 km^2^ where approximately 495,000 people live. The 75 BG sentinel traps were positioned at the southern limit of *w*Mel deployments including regions with and without mosquito releases. A total of 30,786 *Culex* spp. were trapped (61.28%), followed by 19,427 *Ae. aegypti* (38.67%), of which 7,613 were individually screened for *Wolbachia* presence, and 27 *Ae. albopictus* (0.05%). Both *Ae. aegypti* and *Culex* spp. showed seasonal population fluctuations, although a decrease in the catches of both taxa were recorded between March to May 2022, a period with intense dengue transmission, and correspondingly more intense vector control efforts (S1 Fig).

### *Wolbachia* frequency over time

**Fig 2:**
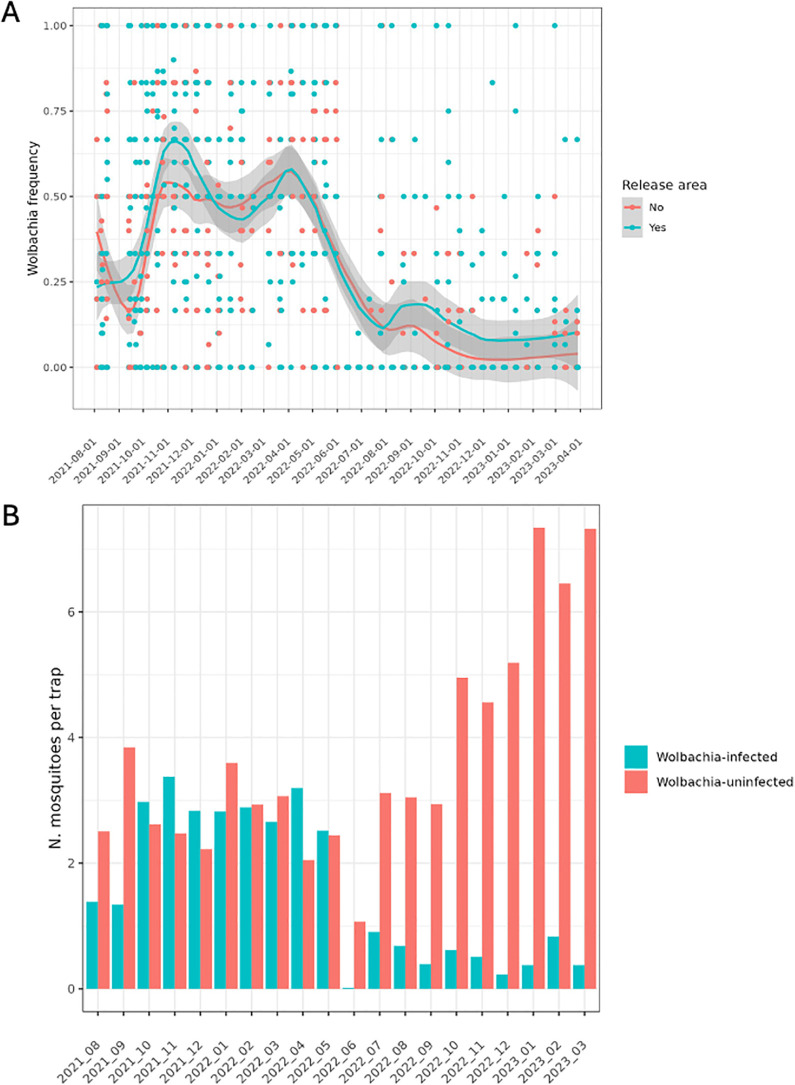
Number and frequencies of *Wolbachia*-infected and -uninfected *Ae. aegypti* over time in Rio de Janeiro. (A) The frequency over time of *Wolbachia*-infected mosquitoes aggregated over neighborhoods with and without *w*Mel releases. The dots represent observations per neighborhood. The blue color indicates areas with *Wolbachia* releases and the red color those without releases. The curve and shaded areas indicate a smoothed curve constructed with these observations over time. (B) The average number of mosquitoes captured fortnightly per trap over the study period in all neighborhoods. *w*Mel deployment: August 2017 –December 2019; Monitoring: August 2021 – March 2023.

**Fig 3:**
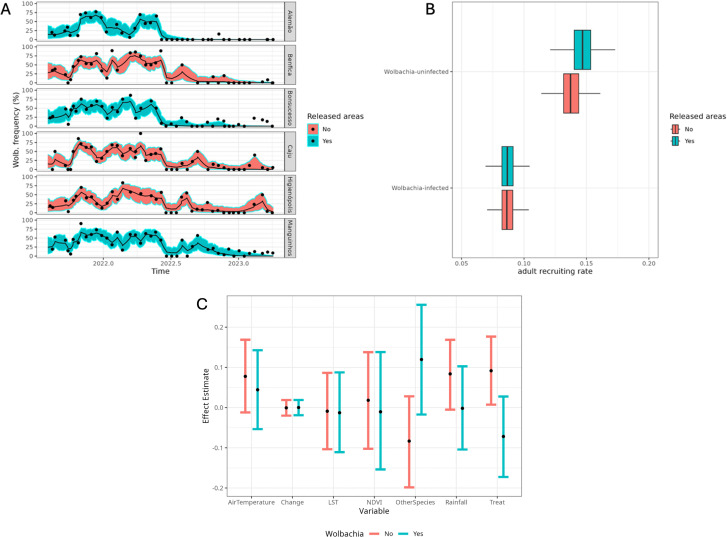
Wolbachia atributes during the monitoring period in Rio de Janeiro. (A) The trajectories of *Wolbachia* frequency (%) over time as evaluated by the model. Points represent observations used for the inference, whereas the lines and shaded areas indicate the mean values and credibility intervals, respectively, given by the model. (B) The basic adult recruiting rate (x-axis) as inferred from the statistical model on number of mosquitoes per day. This variable excludes the effects of external variables such as environmental variables. The areas (color blue and red) depict density distributions from the MCMC simulations. The boxplots distinguish the distributions inferred for the parameter estimation for *Wolbachia*-uninfected mosquitoes (NW) and *Wolbachia*-infected mosquitoes (W); (C) Impact of external variables - air temperature, change to spinosad (lagged by 4 months), land temperature (LST), NDVI, presence of other mosquitoes, rainfall - as inferred from the MCMC simulations. The colors indicate *Wolbachia*-infected and -uninfected *Ae. aegypti* mosquitoes. NDVI is the index of vegetation coverage. ‘*OtherSpecies*’ refer to the proportion of species collected at traps, other than *Aedes aegypti* mosquitoes. The effect is a multiplicative factor (no unit) and a zero value for the y-axis indicates no effect.

Both the areas where *Wolbachia* had been released and without releases evidenced similar overall frequencies of *w*Mel over time (Fig 2A), highlighting initial invasion and maintenance of mosquitoes within the release areas, but also its expansion to neighboring areas of deployments. The *w*Mel frequency over the 20 months showed two different patterns. First, from August 2021 to May 2022 the *Wolbachia* frequency ranged between 20–55% in the field collected *Ae. aegypti*. Then, a significant decline in *w*Mel frequency started around May 2022 and lasted until August 2022. By that stage, the *Wolbachia* frequency remained below 10% in both *w*Mel released and non-released areas until the end of our monitoring period in March 2023 (Fig 2A). The *w*Mel frequency was erratic over the first two months of observation (Aug-Sep 2021), fluctuating between 20 and 40% (Fig 2A), but the number of both *Wolbachia*-infected and -uninfected mosquitoes remained stable from October 2021 to May 2022, with a *w*Mel frequency around 50%. In June 2022, the number of *Ae. aegypti* dropped dramatically, with <1 *Wolbachia*-uninfected mosquitoes and near zero *Wolbachia*-infected mosquitoes/trap. The number of *Wolbachia*-uninfected mosquitoes returned to ~3 mosquitoes/trap in the next month with a twofold increase by 2023 (>6 mosquitoes/trap) (Fig 2B). Despite this increase, *Wolbachia*-infected mosquitoes declined to less than 10% frequency in the field during the last six months of observation (Fig 2A). It is noteworthy that the decline in *Wolbachia* frequency was not a localized phenomenon, but in all monitored neighborhoods (Fig 3A). The basic adult recruitment rate of *Wolbachia*-uninfected mosquitoes was significantly higher than of *Wolbachia*-infected mosquitoes, indicating a better capacity of uninfected mosquitoes to recover after a sharp population decline (Fig 3B). Mean rates of adult recruitment for the uninfected mosquitoes in *Wolbachia* released and non-released areas were 0.15 (CI: 0.13 - 0.17) and 0.14 (CI: 0.12 - 0.16), respectively, while the rate for infected mosquitoes was 0.09 (CI: 0.07 - 0.10) when considered across both areas (Fig 3B).

### Influence of external variables on *Wolbachia* frequency

We inferred a possible influence of biological and environmental variables, as well as chemical interventions on both *w*Mel-infected and -uninfected *Ae. aegypti* mosquito frequencies in the field (Fig 3C). The 95% credibility intervals tended to overlap with 0 and to overlap between the *w*Mel infected and uninfected mosquitoes. However, the presence of other mosquito species and insecticide treatment may have had opposite effects for *w*Mel-infected and *w*Mel-uninfected mosquitoes on adult recruitment rate (Fig 3C). The presence of other mosquitoes (*Culex* sp. and *Ae. albopictus*) was negatively correlated with *w*Mel-uninfected mosquitoes (mean = -0.08 CrI: -0.20 – 0.03) but was positively correlated with *w*Mel-infected mosquitoes (mean = 0.12 CrI: -0.02 – 0.26). The insecticide treatment led to a higher recruitment rate for *w*Mel-uninfected mosquitoes (mean = 0.09 CrI: 0.01 – 0.18) than for *w*Mel-infected mosquitoes (mean = -0.07 CrI: -0.17 – 0.03). Rainfall had a positive association with *w*Mel-uninfected mosquitoes (mean = 0.08 CrI: -0.01 – 0.17) but was not associated with *w*Mel-infected mosquitoes (mean 0.0 CrI: -0.10 – 0.10). Air and land temperatures, and NVDI did not have significant effects on mosquitoes (Fig 3C).

### Spatial modelling

**Fig 4:**
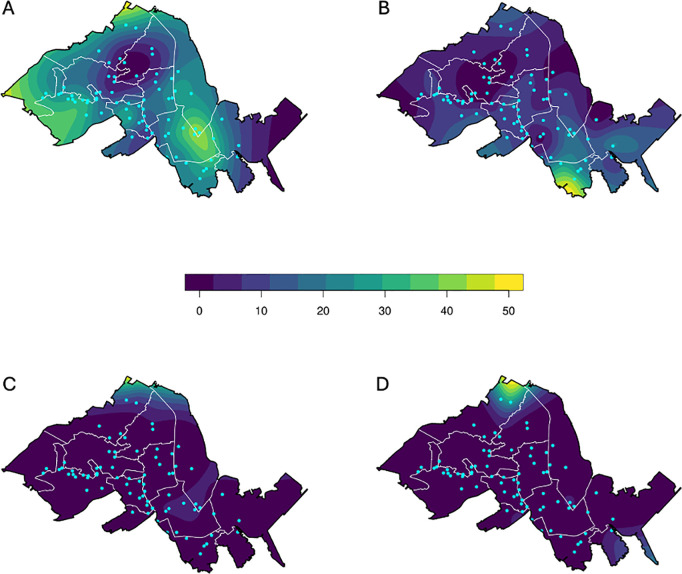
Generalized Additive Model (GAM) fitted to the proportion of *w*Mel-infected *Ae. aegypti* mosquitoes collected in the BG-Sentinel traps spread over our study area for each of the five-month periods. The legend represents the frequency of wMel in the landscape. A = the period between Aug.2021-Dec.2021, B = Jan.2022-May.2022, C = Jun.2022-Oct.2022, and D = Nov.2022-March.2023. *w*Mel deployment: August 2017 –December 2019; Monitoring: August 2021 – March 2023. The base layer map was downloaded from the open-source site of IBGE: https://www.ibge.gov.br/geociencias/downloads-geociencias.html.

After assessing the temporal variation in *w*Mel frequency (Figs 2 and 3), we examined it spatially (Table 1, Fig 4). The spatial component was significant in all periods, reinforcing the significant role of the spatial term in the model during the 20-months period (Table 1). In period A, there was a higher *w*Mel density in the neighborhoods with previous deployment, or very close to those regions. In period B, a high-frequency area was only noticeable in the south, but in the periods C and D, both after a reduction in *w*Mel frequency, only a small northern area maintained a relatively higher infection frequency. This effect, however, may be attributed to edge effects in the smoothing function used.

**Table 1:** Results of the Generalized Additive Model (GAM) with binomial family fitted to the proportion of *w*Mel *Aedes aegypti* mosquitoes with the trap location (whether in a previous deployment zone) as a co-variate.

Period	A			B			C			D		
	β	95% CI	P-value	β	95% CI	P-value	β	95% CI	P-value	β	95% CI	P-value
*w*Mel-release (Yes)	0.4	0.15 - 0.65	**0.002**	0.33	0.02 - 0.64	**0.038**	-0.27	-0.97 - 0.42	0.4	0.65	-0.33 - 1.6	0.2
Spatial term s(x,y)			**<0.001**			**<0.001**			**<0.001**			**<0.001**
AIC	455			416			236			199		
Deviance	108			110			82.6			43.5		
No. Obs	73			67			65			61		

### Loss of *Wolbachia* in field *Aedes aegypti* mosquitoes

**Fig 5:**
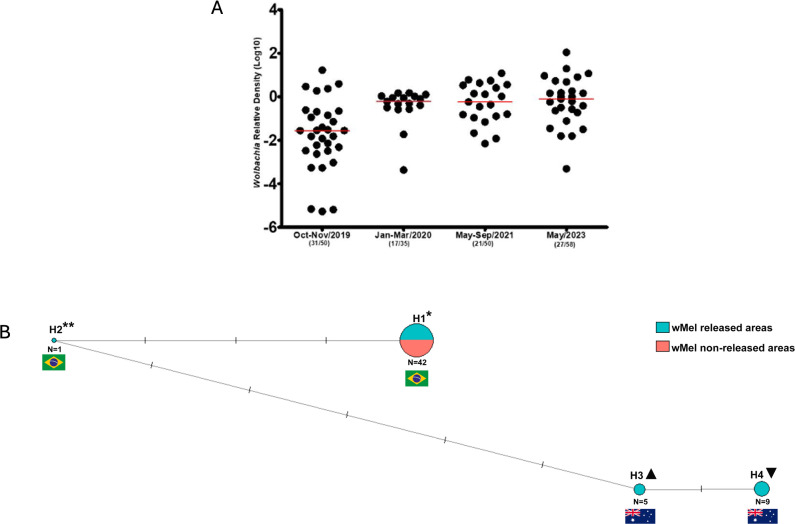
*Wolbachia* density and mtDNA status of uninfected mosquitoes in Rio de Janeiro. (A) Relative density of *w*Mel strain from field-caught mosquitoes between 2019-2023, (B) Network analysis of COI sequences of *Ae. aegypti* mosquitoes PCR-negative for wMel, showing that 24.56% of mosquitoes had the ancestral mtDNA of Australian *Ae. aegypti* populations. The bars on the lines represent mutational steps. The number of sequences in each haplotype does not include reference sequences. Reference sequences: H1* - Brazil (GenBank accession KU936162, JX456411); H2** – Colombia, South America (KM203142, KM203172); H3▲ – Townsville, Australia (GQ143718); H4▼– Yorkeys Knob, Australia (OM214532). Their positions in the network are evidenced with country flags (Brazil, Colombia, and Australia).

The density of *Wolbachia* in field-caught mosquitoes was evaluated in different seasons throughout four years (2019–2023) and overall did not seem to decrease after deployments (Fig 5A). Indeed, the bacterial density in October-November 2019, when releases have just ceased, was lower than the density observed in the following years. During the last year of analysis, we randomly selected 57 samples collected in summer 2023 in both *Wolbachia* released and non-released areas that were PCR-negative for the bacterium to evaluate potential adverse effects of high temperatures on *w*Mel infection status. Network analysis showed that 14 PCR-negative samples for *Wolbachia* clustered with Australian *Ae. aegypti* COI sequences, and the other 43 were grouped with sequences from the other two clades that represent Brazilian and other South American samples. This points to mosquitoes that have lost *Wolbachia* contributing to 24.56% of uninfected mosquitoes in the field (Fig 5B, S2 Table).

### Insecticide resistance

**Fig 6:**
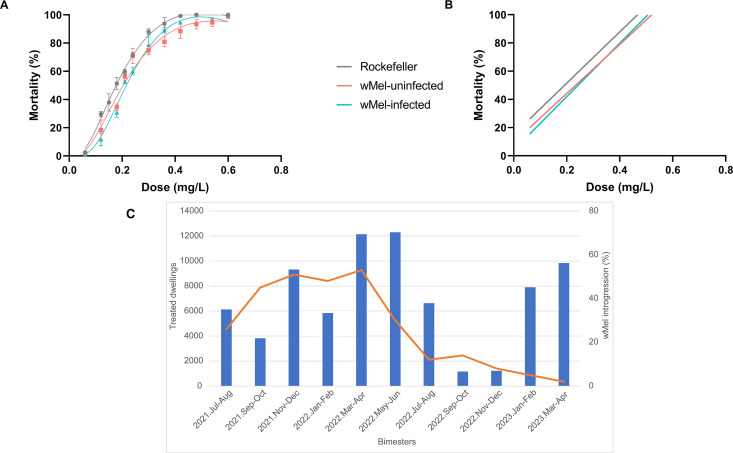
Biological assays for spinosad and *Aedes aegypti* mosquitoes and the field application of spinosad by Rio de Janeiro public health department. (A) Spinosad mortality in a *Wolbachia*-infected, -uninfected, and the Rockefeller *Ae. aegypti* strains. The susceptible strain Rockefeller was used as an internal quality control and an insecticide susceptible reference lineage; (B) Linear regression obtained by exposing larvae of the Urca, *w*Mel and Rockefeller strains to the larvticide spinosad; (C) Total number of dwellings treated with spinosad (bars) and percentage of *w*Mel introgression in field *Ae. aegypti* population (red line). *w*Mel deployment: August 2017 –December 2019; Monitoring: August 2021 – March 2023.

Biological assays identified high levels of susceptibility of both *Wolbachia*-infected and uninfected *Ae. aegypti* mosquitoes to spinosad. The spinosad LCs were similar to the values obtained for the reference Rockefeller strain. The RR_95_ for *Wolbachia*-infected -uninfected mosquitoes were 1.2 with 95% CI 1.0-1.4 and 1.4 with 95% CI 1.1-1.8, respectively, confirming the susceptibility of these populations to the larvicide (Fig 6A, S3 Table). Linear regression analysis indicated similar slopes between *Wolbachia*-infected and -uninfected field mosquitoes and the Rockefeller strain, suggesting that a similar range of doses is required to affect all individuals, i.e., a very homogeneous population response to this larvicide (Fig 6B). Linear regression was utilized to examine the level of heterogeneity among populations concerning the Rockefeller strain. Our data suggest both *w*Mel-infected and *w*Mel-uninfected mosquitoes exhibit similar response to the Spinosad toxicity. Between March-June, when dengue transmission peaks in Rio de Janeiro, the number of spinosad treated dwellings reached 12,000 units, which corresponds to 32% of the total larvicide applied during the 20 months period (Fig 6C).

We estimated the frequency of *kdr* alleles as a proxy for pyrethroid resistance and evaluated the susceptibility of mosquitoes to the larvicide spinosad, due to the distinct impact of the insecticide treatment observed for *Wolbachia*-infected and -uninfected mosquitoes. The *kdr* genotyping showed that there was a similar pyrethroid resistance genotype profile in terms of *kdr* mutations for *Wolbachia*-infected and uninfected mosquitoes. Since the substitutions at positions 1016 (Na_V_R2 allele) and 1534 (Na_V_R1 allele) are recessive—meaning that pyrethroid resistance is only expressed in homozygous individuals—most of the analyzed mosquitoes carried kdr alleles and exhibited resistant genotypes (R1R1, R1R2, or R2R2) (S4 Table, S2 Fig).

## Discussion

The deployment of *Aedes aegypti* mosquitoes infected with *Wolbachia* represents a promising strategy for mitigating arbovirus transmission, as evidenced by multiple reports of decreased dengue transmission in cities across Australia, Colombia, Indonesia, and Vietnam, where the program advanced [19–21,25,49]. In Brazil, however, contrasting epidemiological outcomes have been observed between the neighboring cities of Rio de Janeiro and Niterói, despite being separated only by a 14 km bridge [24,25]. In Niterói, releases began in February 2017 and continued until December 2019, covering an area of 40 km^2^. The establishment of *w*Mel was heterogeneous across release zones, with median prevalence ranging from 40 to 90%, which was associated with an overall 69% reduction in dengue transmission [24]. In contrast, Rio de Janeiro experienced a milder reported reduction in dengue transmission, despite geographical proximity and similar environmental and climatic conditions. In Rio, releases occurred between August 2017 and December 2019, covering 86.8 km^2^, equivalent to 13.55% of the city’s urban area. However, *w*Mel frequency often dropped below 30%, particularly during the warmer months when dengue transmission typically peaks. Concomitantly, a reduction of 38% and 10% was reported in the incidence of DENV and CHIKV, respectively [25]. In this study, we independently sampled > 50,000 adult mosquitoes over a 20-month period to investigate whether *w*Mel introgression would expand or contract following the cessation of *Wolbachia* deployments in Rio in 2019. Our independent evaluation suggests that an overall population crash in mosquito populations in Rio de Janeiro followed the arrival of a new larvicide, with a failure of *w*Mel frequency to recover subsequently. These findings emphasize the need of a better integration with complementary vector control approaches under an Integrated Vector Management (IVM) strategy.

Both *w*Mel-infected and -uninfected mosquitoes had a high susceptibility to spinosad and high resistance to pyrethroids as demonstrated by in vivo and molecular assays, ruling out the occurrence of the same problem observed in the first deployments in Brazil [18,23]. Previous data from our group showed that in 2015, after 20 weeks of consecutive releases, the frequency of *w*Mel decreased significantly in the isolated community of Tubiacanga. This was attributed to the ongoing and widespread use of pyrethroids by local households for personal protection against mosquito nuisance and female biting during local deployment [18]. This time, the independent monitoring started 20 months after *w*Mel releases ceased, in August 2021 when there would have been ample time to break down any initial disequilibrium between the *Wolbachia* infection and the nuclear genome where resistance alleles are located [50]. Nevertheless, the *w*Mel frequency remained stable until April-May 2022, then dropped significantly and simultaneously in all the 12 neighborhoods analyzed in this study (Figs 2 and 5).

The public health department of Rio de Janeiro switched from the pyriproxyfen larvicide to the biolarvicide spinosad when we started this study, in August 2021. The distribution of this new compound throughout the city and the training of health agents to effectively distribute it would have started at a slow pace. Dengue transmission in Rio often peaks between April and May [51], exactly when the number of spinosad treated dwellings peaked and reached 32% of the total application during the 20 months period (Fig 6C). Therefore, it was anticipated (and observed) that mosquito population density of both *w*Mel-infected and -uninfected mosquitoes were strongly reduced by spinosad during the peak of dengue transmission in the city.

The number, and consequently, the proportion of captured *w*Mel-infected mosquitoes remained low up to the end of monitoring (<10%) after the mosquito population crash, whereas the *w*Mel-uninfected mosquitoes recovered soon to levels even higher than before and fluctuated seasonally as expected. We hypothesized that the main explanation for the increase of only the *w*Mel-uninfected mosquitoes relates to the fitness cost associated with this *Wolbachia* strain on mosquito life-history traits [52]. It is well known that when infected with *w*Mel, *Ae. aegypti* eggs present a much lower resistance to desiccation when compared to uninfected counterparts [53]. After 12 weeks of egg storage, an egg hatch rate of 45% egg-hatching was observed for *w*Mel-uninfected mosquitoes, whereas only 4.8% of *w*Mel-infected eggs hatched, i.e., a 10x worst rate for *w*Mel-infected mosquitoes [54]. Under field conditions, a likely consequence of the low egg-hatching of *Wolbachia*-infected eggs is the absence of a substantial egg bank, a repository for *Ae. aegypti* populations. The presence of an egg bank is traditionally viewed as an evolutionary response to overwintering or a dry season, allowing a synchronous hatching of *Ae. aegypti* eggs with the return of environmentally suitable conditions [55–57]. With the disruption of the adult mosquito population (as seen in the reduction of catches in the BG-Traps), the bigger egg bank available for the *w*Mel-uninfected mosquitoes would be expected to lead to the reestablishment of uninfected mosquitoes, whereas the *w*Mel-infected mosquitoes should show a lower recruitment rate, which was estimated to represent a 40% reduction (Fig 4).

Another potential factor decreasing the *w*Mel-infected mosquito population in the field is the heat sensitivity of this bacterial strain, which is well described in the literature [31]. High temperatures impact *w*Mel relative density and maternal transmission efficiency both in laboratory and field conditions, as observed in Australia and Vietnam [32,58–61]. Under intense heatwaves or longer periods of high temperatures in breeding sites, the *w*Mel strain may be lost. Based on a multi-country assessment, the observed loss of *w*Mel was heterogeneous, ranging from 0 to 20% in five different countries where deployments took place [62]. We sampled adult *Ae. aegypti* during Brazilian summer (Jan-Feb/2023) and estimated a loss of the infection of 24.5% based on mtDNA data. Assuming high temperature is a key factor in *w*Mel loss, it is likely that seasonal variation could contribute to the fluctuation in introgression as demonstrated in Rio de Janeiro [25], although the fitness differences noted above for egg viability would further exacerbate any differences in frequency. Notably, a previous study revealed a 50% *w*Mel frequency in Rio de Janeiro during winter, but a reduction to 25% in warmer months [25], which is coincident with the period of dengue transmission in the city [51]. Nevertheless, the heat sensitivity of *w*Mel was not responsible for the population crash observed in this study, since both *w*Mel-infected and -uninfected mosquitoes were impacted during the cooler periods in Rio.

We collected very few *Ae. albopictus*, making difficult to test any hypothesis regarding possible competition with *Ae. aegypti* or population replacement, as previously described [63–65]. Considering the landscape and the highly urbanized areas that mosquitoes were sampled, the *Culex* spp. we sampled were most likely *Cx. quinquefasciatus*. Surprisingly, the presence of *Culex* spp. seemed to positively affect *w*Mel-infected *Ae. aegypti*. We do not have a plausible hypothesis for this observation, since interactions between *Ae. aegypti* and *Cx. quinquefasciatus* are unexpected due to the distinct biology of these species, which rarely share the same breeding sites and have different periods of feeding activity [66].

Our study has limitations that must be acknowledged. The sampling network and the BG-Sentinel trap placements did not cover all the original released zones of *Ae. aegypti* infected with *w*Mel. We focused our trapping on an area of the city in which there were a cluster of dangerous Brazilian favelas and monitoring is harder to follow up. Our sampling efforts were based on 75 BG-Traps across an area of 33.5 Km^2^, which is similar to the trap density adopted by WMP to monitor introgression post-deployment, but low when assessing finer spatial patterns. Since we were able to screen only a fraction of captured *Ae. aegypti* mosquitoes (39.2%), we acknowledge lower accuracy of *Wolbachia* frequency estimates than in some other sudies.

IVM represents a strategic, evidence-based framework designed to optimize resource utilization in vector control efforts. This approach necessitates a comprehensive management strategy that enhances the efficacy, cost-effectiveness, ecological integrity, and sustainability of vector control interventions by leveraging available tools and resources [67]. It is well known that vector control interventions rely heavily on the use of insecticides, one cornerstone of the IVM approach advocated by the WHO [68]. Since the development of insecticide resistance could undermine vector control efforts, insecticide sequence, rotation and mixture have been recommended to avoid the permanent product discontinuity for public health usage. Since 2001, Brazilian Ministry of Health has recommended the switch of larvicide five times, i.e., an average of one rotation every 4.5 years. Therefore, as *w*Mel deployment areas are expanding globally, the combined use of insecticides and *Wolbachia* must be planned and implemented in a coordinated manner preferentially under the supervision of national and local authorities. Otherwise, *Wolbachia* frequency could drop and eventually crash with the introduction of a new insecticide, limiting its application on city- and nationwide public health interventions.

One question that arises from our results is how to integrate insecticide application with *Wolbachia* deployments, given that they rely on opposing strategies: suppression *versus* invasion and replacement. Higher dengue blockage is achieved in situations where introgression is relatively more successful [68,69]. Hence, to provide a long-term sustainable *Wolbachia* introgression and, by corollary, maximize the *Wolbachia*-based effect on dengue transmission, insecticide applications within dengue endemic cities must consider *Wolbachia* status over the treated areas. To avoid jeopardizing the introgression of *Wolbachia*, public health teams should avoid or limit the use of insecticides at the onset of releases. Based on the epidemiological endpoint claimed in Niteroi, a city apart from Rio by a 14Km bridge and so with similar environmental conditions, a stronger dengue transmission reduction in Rio de Janeiro (around 55–80%) would rely on achieving a higher *Wolbachia* frequency on the field, as seen in Niteroi (around 70%. Introgression).

This study underscores the critical importance of sustained monitoring and evaluation of *Wolbachia* frequency in field *Ae. aegypti* populations to assess the potential reduction of arbovirus transmission and ensure long-term protection in targeted areas. In Brazil, the WMP has concluded citywide deployments in five Brazilian cities since 2017, but results are available for only two of them, Rio de Janeiro and Niterói. Notwithstanding, an additional expansion was announced for six more cities spread over the country [70]. We strongly recommend that active surveillance of *Ae. aegypti* and *Wolbachia* introgression in deployed areas be carried out and evaluated by independent teams on a timely manner. Such independent evaluation would provide (i) an autonomous and credible assessment of the performance of *Wolbachia*, (ii) input to help improving its effectiveness through feedback of lessons learned, and (iii) support local public health teams to optimize vector control interventions.

Our results have shown that the long-term persistence of *Wolbachia* after introgression in dengue endemic areas can be uncertain. The sustainability of *w*Mel in high transmission settings is questionable. The *w*Mel history in Rio de Janeiro involves one failure due to pyrethroids use in Tubiacanga and the recent population wide crash on a much larger geographic extension of the city after the larvicide was switched to spinosad. Independent evaluation of *Wolbachia* introgression is necessary to provide transparency and accountability for local communities hosting deployments and local government that support this strategy with funds and personnel. *Wolbachia* releases need to be undertaken under an IVM program to optimize efforts on dengue mitigation. There is more than meets the eye about the adoption of *Wolbachia* as a control strategy – it is complex to introgress *Wolbachia* and its persistence requires continuous monitoring and releases. It becomes essential to implement technical guidance on IVM in Brazil prior the nationwide release of *Wolbachia*-infected mosquitoes in order to optimize dengue mitigation efforts while ensuring the judicious use of resources [71].

## Supporting information

S1 Table Sociodemographic data of the 12 neighborhoods where BG-Sentinel traps were fortnightly inspected. HDI refers to the Human Development Index.
(DOCX)

S2 Table *Aedes aegypti* mosquitoes PCR-negative for *Wolbachia* that had a 866-bp fragment of the mitochondrial COI sequenced in this study. Sequences from Rio de Janeiro, Brazil, Colombia/Bolivia, and Australia were used as control. South American haplotypes were 1 and 2, while Australian haplotypes were 3 and 4. * All samples used in this study were collected in Rio de Janeiro, Brazil and thus the neighbourhood names are detailed.
(DOCX)

S3 Table Toxicity of spinosad against 3rd instar *Aedes aegypti* larvae after 24h exposure. LC = Lethal concentration (mg/L), CI 95% = Confidence interval 95%, RR = Resistance ratio.
(DOCX)

S4 Table Numbers and frequencies of *Wolbachia*-infected and -uninfected *Ae. aegypti* mosquitoes observed per resistance genotype. Mosquitoes were sampled in the field using BG-Sentinel traps. Allele without mutation = S, allele with a mutation only at site 1534 = R1, and allele with mutations at both site 1016 and site 1534 = R2.
(DOCX)

S1 Fig Baseline entomological and epidemiological information from Rio de Janeiro during our study period. (A) Seasonal variation in *Aedes aegypti* and *Culex* spp. in the fortnightly trapping in Rio de Janeiro, (B) Dengue incidence in Rio de Janeiro, calculated per 100,000 inhabitants.
(DOCX)

S2 Fig Frequency of the knockdown resistance (*kdr*) genotypes in field *Wolbachia*-infected and -uninfected *Aedes aegypti* captured in Rio de Janeiro in February and March 2023. (DOCX)
