# Supplementary material for: The long-term persistence of the wMel strain in Rio de Janeiro is threatened by poor integrated vector management and bacterium fitness cost on Aedes aegypti
Source: PLoS Negl Trop Dis. 2025 Jul 23;19(7):e0013372. doi: 10.1371/journal.pntd.0013372 (PMC12310003; doi:10.1371/journal.pntd.0013372)
Supplement: S1 Table — HDI refers to the Human Development Index. (DOCX) [file pntd.0013372.s001.docx]

**Table S1.** Sociodemographic data of the 12 neighborhoods where BG-Sentinel traps were fortnightly inspected. HDI refers to the Human Development Index.

| **Neighborhood** | **Code** | ***Wolbachia* release** | **Socio-demographic description** | **Inhabitants (2000)** | **Houses (2010)** | **Human density per house** | **Area (Km^2^, 2003)** | **HDI (2000)** |
| --- | --- | --- | --- | --- | --- | --- | --- | --- |
| Benfica | A | No | lower middle class | 25081 | 7778 | 3.22 | 1.74 | 0.761 |
| Caju | B | No | lower middle class | 20477 | 6481 | 3.16 | 5.35 | 0.692 |
| Engenho da Rainha | C | No | lower middle class | 26659 | 8938 | 2.98 | 2.23 | 0.794 |
| Higienópolis | D | No | suburban | 15734 | 6327 | 2.49 | 1.16 | 0.823 |
| Inhaúma | E | No | favela (slum) | 45698 | 15046 | 3.04 | 3.49 | 0.764 |
| Maria da Graça | F | No | working class | 7972 | 2820 | 2.83 | 0.86 | 0.781 |
| Bonsucesso | G | Yes | lower middle class | 24315 | 8428 | 2.89 | 2.24 | 0.817 |
| Complexo do Alemão | H | Yes | favela (slum) | 69143 | 21025 | 3.29 | 2.96 | 0.657 |
| Manguinhos | I | Yes | favela (slum) | 36610 | 10565 | 3.47 | 2.68 | 0.674 |
| Maré | J | Yes | favela (slum) | 129770 | 41731 | 3.11 | 4.27 | 0.667 |
| Olaria | K | Yes | suburban | 57514 | 19827 | 2.90 | 3.69 | 0.801 |
| Ramos | L | Yes | suburban | 35188 | 12294 | 2.86 | 2.79 | 0.801 |
