# Supplementary material for: The long-term persistence of the wMel strain in Rio de Janeiro is threatened by poor integrated vector management and bacterium fitness cost on Aedes aegypti
Source: PLoS Negl Trop Dis. 2025 Jul 23;19(7):e0013372. doi: 10.1371/journal.pntd.0013372 (PMC12310003; doi:10.1371/journal.pntd.0013372)
Supplement: S2 Table — Sequences from Rio de Janeiro, Brazil, Colombia/Bolivia, and Australia were used as control. South American haplotypes were 1 and 2, while Australian haplotypes were 3 and 4. * All samples used in this study were collected in Rio de Janeiro, Brazil and thus the neighbourhood names are detailed. (DOCX) [file pntd.0013372.s002.docx]

**Table S2**. ***Aedes aegypti* mosquitoes PCR-negative for *Wolbachia* that had a 866-bp fragment of the mitochondrial COI sequenced in this study.** Sequences from Rio de Janeiro, Brazil, Colombia/Bolivia, and Australia were used as control. South American haplotypes were 1 and 2, while Australian haplotypes were 3 and 4.

| **Sample** | **Locality*** | **Sampling Date** | ***Wolbachia* released area** | **Haplotype** | **GenBank** |
| --- | --- | --- | --- | --- | --- |
| BG2M1 | Engenho da Rainha | 2023-01-25 | No | 3 | PQ424073 |
| BG3M2 | Inhaúma | 2023-02-06 | No | 1 | PQ424051 |
| BG11F2 | Inhaúma | 2023-01-25 | No | 3 | PQ424074 |
| BG11F3 | Inhaúma | 2023-01-25 | No | 3 | PQ424075 |
| BG16M3 | Alemão | 2023-01-11 | Yes | 3 | PQ424076 |
| BG23M6 | Alemão | 2023-01-11 | Yes | 1 | PQ424077 |
| BG23M2 | Alemão | 2023-01-11 | Yes | 4 | PQ424052 |
| BG23M7 | Alemão | 2023-01-11 | Yes | 1 | PQ424053 |
| BG25M1 | Benfica | 2023-02-28 | No | 1 | PQ424078 |
| BG30M1 | Benfica | 2023-01-10 | No | 1 | PQ424054 |
| BG31M3 | Caju | 2023-02-28 | No | 1 | PQ424079 |
| BG34M2 | Caju | 2023-01-10 | No | 1 | PQ424055 |
| BG34M2 | Caju | 2023-01-24 | No | 1 | PQ424056 |
| BG34M6 | Caju | 2023-01-24 | No | 1 | PQ424057 |
| BG34F1 | Caju | 2023-02-28 | No | 1 | PQ424080 |
| BG34M7 | Caju | 2023-02-28 | No | 1 | PQ424068 |
| BG34M4 | Caju | 2023-01-24 | No | 1 | PQ424081 |
| BG35M3 | Manguinhos | 2023-02-28 | Yes | 2 | PQ424082 |
| BG35M5 | Manguinhos | 2023-02-28 | Yes | 1 | PQ424083 |
| BG38M2 | Manguinhos | 2023-02-07 | Yes | 1 | PQ424058 |
| BG38M1 | Manguinhos | 2023-02-07 | Yes | 1 | PQ424084 |
| BG41M1 | Manguinhos | 2023-02-07 | Yes | 1 | PQ424085 |
| BG41M5 | Manguinhos | 2023-02-28 | Yes | 4 | PQ424086 |
| BG41M6 | Manguinhos | 2023-02-28 | Yes | 4 | PQ424087 |
| BG41M2 | Manguinhos | 2023-01-11 | Yes | 1 | PQ424059 |
| BG41M4 | Manguinhos | 2023-02-28 | Yes | 4 | PQ424069 |
| BG45F6 | Maré | 2023-02-28 | Yes | 1 | PQ424088 |
| BG48M4 | Maré | 2023-02-24 | Yes | 4 | PQ424060 |
| BG48F7 | Maré | 2023-01-24 | Yes | 4 | PQ424089 |
| BG48F8 | Maré | 2023-01-24 | Yes | 4 | PQ424090 |
| BG48M1 | Maré | 2023-01-24 | Yes | 1 | PQ424091 |
| BG49F1 | Maré | 2023-01-10 | No | 1 | PQ424092 |
| BG49M1 | Maré | 2023-02-28 | No | 1 | PQ424093 |
| BG49F2 | Maré | 2023-01-10 | No | 1 | PQ424061 |
| BG49M4 | Maré | 2023-02-28 | No | 1 | PQ424062 |
| BG50F1 | Olaria | 2023-01-10 | No | 1 | PQ424094 |
| BG50M2 | Olaria | 2023-01-10 | No | 1 | PQ424096 |
| BG50M1 | Olaria | 2023-02-28 | No | 1 | PQ424095 |
| BG52M2 | Olaria | 2023-02-28 | Yes | 1 | PQ424063 |
| BG52M3 | Olaria | 2023-02-28 | Yes | 1 | PQ424064 |
| BG52F8 | Olaria | 2023-02-28 | Yes | 1 | PQ424097 |
| BG52M1 | Olaria | 2023-02-28 | Yes | 1 | PQ424098 |
| BG52M3 | Olaria | 2023-02-06 | Yes | 1 | PQ424099 |
| BG52M4 | Olaria | 2023-02-28 | Yes | 1 | PQ424100 |
| BG52M5 | Olaria | 2023-02-28 | Yes | 4 | PQ424101 |
| BG52M7 | Olaria | 2023-02-06 | Yes | 1 | PQ424102 |
| BG55F3 | Ramos | 2023-02-28 | Yes | 1 | PQ424065 |
| BG55M1 | Ramos | 2023-02-28 | Yes | 3 | PQ424066 |
| BG57M2 | Ramos | 2023-02-28 | Yes | 1 | PQ424070 |
| BG57F1 | Ramos | 2023-02-28 | Yes | 4 | PQ424103 |
| BG57M1 | Ramos | 2023-02-28 | Yes | 1 | PQ424104 |
| BG60M5 | Bonsucesso | 2023-01-10 | Yes | 1 | PQ424105 |
| BG65M5 | Bonsucesso | 2023-02-28 | Yes | 1 | PQ424106 |
| BG69M3 | Higienópolis | 2023-01-11 | No | 1 | PQ424067 |
| BG69M4 | Higienópolis | 2023-01-11 | No | 1 | PQ424071 |
| BG73M5 | Higienópolis | 2023-01-11 | No | 1 | PQ424107 |
| BG73M4 | Higienópolis | 2023-02-06 | No | 1 | PQ424072 |
| *Control* | Pernambuco, Brazil | - | - | 1 | JX456411 |
| *Control* | Rio de Janeiro, Brazil | - | - | 1 | KU936162 |
| *Control* | Colombia/Bolivia | 2012/2013 | - | 2 | KM203142 |
| *Control* | Townsville, Australia | - | - | 3 | GQ143718 |
| *Control* | Yorkeys Knob, Australia | 2018 | - | 4 | OM214532 |

* All samples used in this study were collected in Rio de Janeiro, Brazil and thus the neighbourhood names are detailed.
