# Supplementary material for: The long-term persistence of the wMel strain in Rio de Janeiro is threatened by poor integrated vector management and bacterium fitness cost on Aedes aegypti
Source: PLoS Negl Trop Dis. 2025 Jul 23;19(7):e0013372. doi: 10.1371/journal.pntd.0013372 (PMC12310003; doi:10.1371/journal.pntd.0013372)
Supplement: S3 Table — LC = Lethal concentration (mg/L), CI 95% = Confidence interval 95%, RR = Resistance ratio. (DOCX) [file pntd.0013372.s003.docx]

**Table S3.** Toxicity of spinosad against 3rd instar *Aedes aegypti* larvae after 24h exposure. LC = Lethal concentration (mg/L), CI 95% = Confidence interval 95%, RR = Resistance ratio

| Group | Number of larvae | LC_50_ | LC_50_  CI 95% | RR_50_ | RR_50_  CI 95% | LC_95_ | LC_95_ CI 95% | RR_95_ | RR_50_  CI 95% |
| --- | --- | --- | --- | --- | --- | --- | --- | --- | --- |
| Rockefeller | 2,880 | 0.17 | 0.11 - 0.24 | 1.00 | 0.75 - 1.34 | 0.38 | 0.27 - 0.49 | 1.00 | 0.42 - 2.39 |
| *w*Mel-uninfected | 2,900 | 0.21 | 0.15 - 0.28 | 1.22 | 0.90 -1.66 | 0.53 | 0.41 - 0.65 | 1.39 | 0.47 - 4.06 |
| *w*Mel-infected | 2,960 | 0.21 | 0.16 - 0.27 | 1.23 | 0.90 - 1.68 | 0.46 | 0.37 - 0.55 | 1.20 | 0.47 - 3.09 |
