# Supplementary material for: The long-term persistence of the wMel strain in Rio de Janeiro is threatened by poor integrated vector management and bacterium fitness cost on Aedes aegypti
Source: PLoS Negl Trop Dis. 2025 Jul 23;19(7):e0013372. doi: 10.1371/journal.pntd.0013372 (PMC12310003; doi:10.1371/journal.pntd.0013372)
Supplement: S4 Table — Mosquitoes were sampled in the field using BG-Sentinel traps. Allele without mutation = S, allele with a mutation only at site 1534 = R1, and allele with mutations at both site 1016 and site 1534 = R2. (DOCX) [file pntd.0013372.s004.docx]

**Table S4.** Numbers and frequencies of *Wolbachia*-infected and -uninfected *Ae. aegypti* mosquitoes observed per resistance genotype. Mosquitoes were sampled in the field using BG-Sentinel traps.

| Group | SS | SR1 | SR2 | R1R1 | R1R2 | R2R2 | Others | Total |
| --- | --- | --- | --- | --- | --- | --- | --- | --- |
| *w*Mel-infected | 0 (0) | 3 (6.7) | 2 (4.4) | 4 (8.9) | 18 (40) | 16 (35.6) | 2 (4.5) | 45 |
| *w*Mel-uninfected | 2 (3.8) | 2 (3.8) | 0 (0) | 2 (3.8) | 15 (28.3) | 31 (58.3) | 1 (1.9) | 53 |

Allele without mutation = S, allele with a mutation only at site 1534 = R1, and allele with mutations at both site 1016 and site 1534 = R2
