# Supplementary material for: The long-term persistence of the wMel strain in Rio de Janeiro is threatened by poor integrated vector management and bacterium fitness cost on Aedes aegypti
Source: PLoS Negl Trop Dis. 2025 Jul 23;19(7):e0013372. doi: 10.1371/journal.pntd.0013372 (PMC12310003; doi:10.1371/journal.pntd.0013372)

**Supplementary Figure 1.** Baseline entomological and epidemiological information from Rio de Janeiro during our study period. (A) Seasonal variation in *Aedes aegypti* and *Culex* spp. in the fortnightly trapping in Rio de Janeiro, (B) Dengue incidence in Rio de Janeiro, calculated per 100,000 inhabitants.


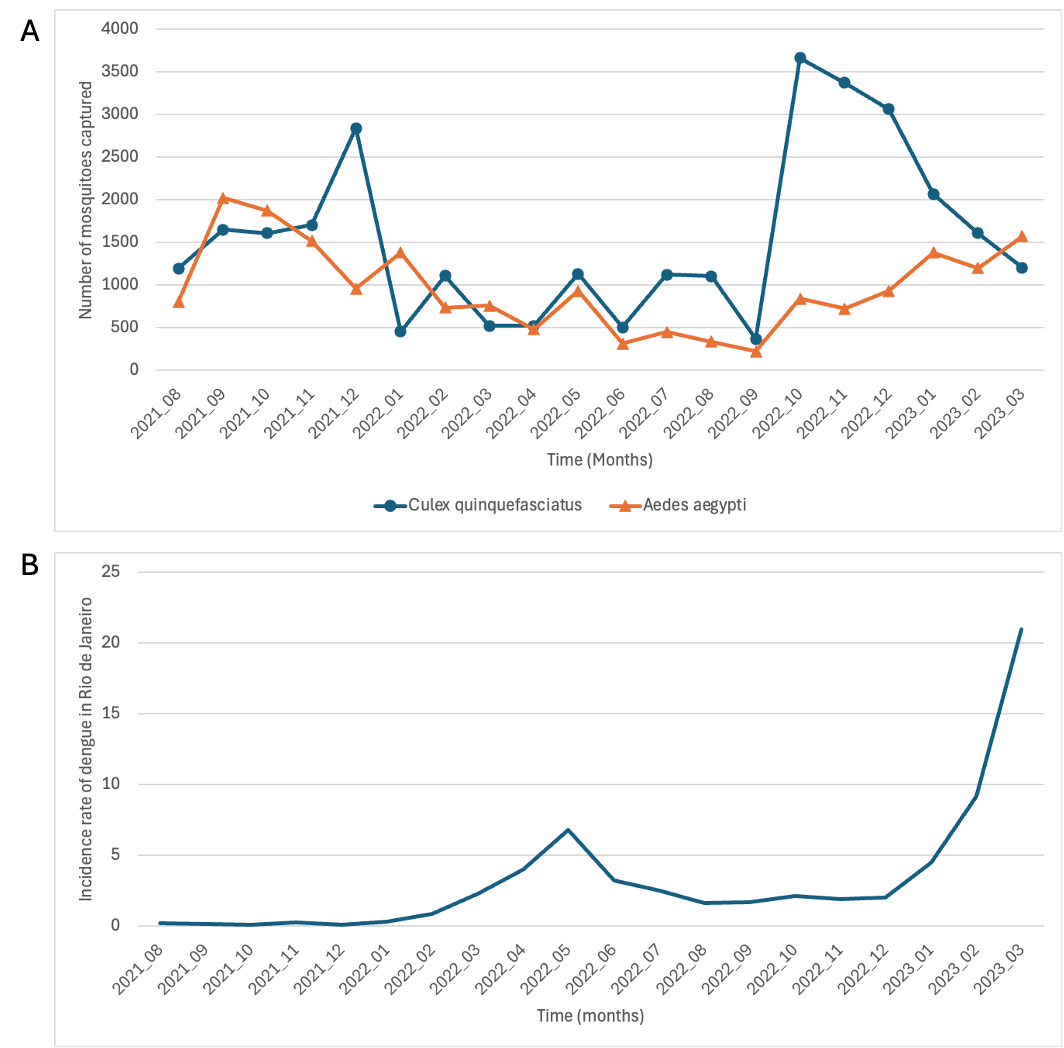

Supplement: S1 Fig — (A) Seasonal variation in Aedes aegypti and Culex spp. in the fortnightly trapping in Rio de Janeiro, (B) Dengue incidence in Rio de Janeiro, calculated per 100,000 inhabitants. (DOCX) [file pntd.0013372.s005.docx]
