# Supplementary material for: The long-term persistence of the wMel strain in Rio de Janeiro is threatened by poor integrated vector management and bacterium fitness cost on Aedes aegypti
Source: PLoS Negl Trop Dis. 2025 Jul 23;19(7):e0013372. doi: 10.1371/journal.pntd.0013372 (PMC12310003; doi:10.1371/journal.pntd.0013372)

**Supplementary Figure 2.** Frequency of the knockdown resistance (*kdr*) genotypes in field *Wolbachia*-infected and -uninfected *Aedes aegypti* captured in Rio de Janeiro in February and March 2023.


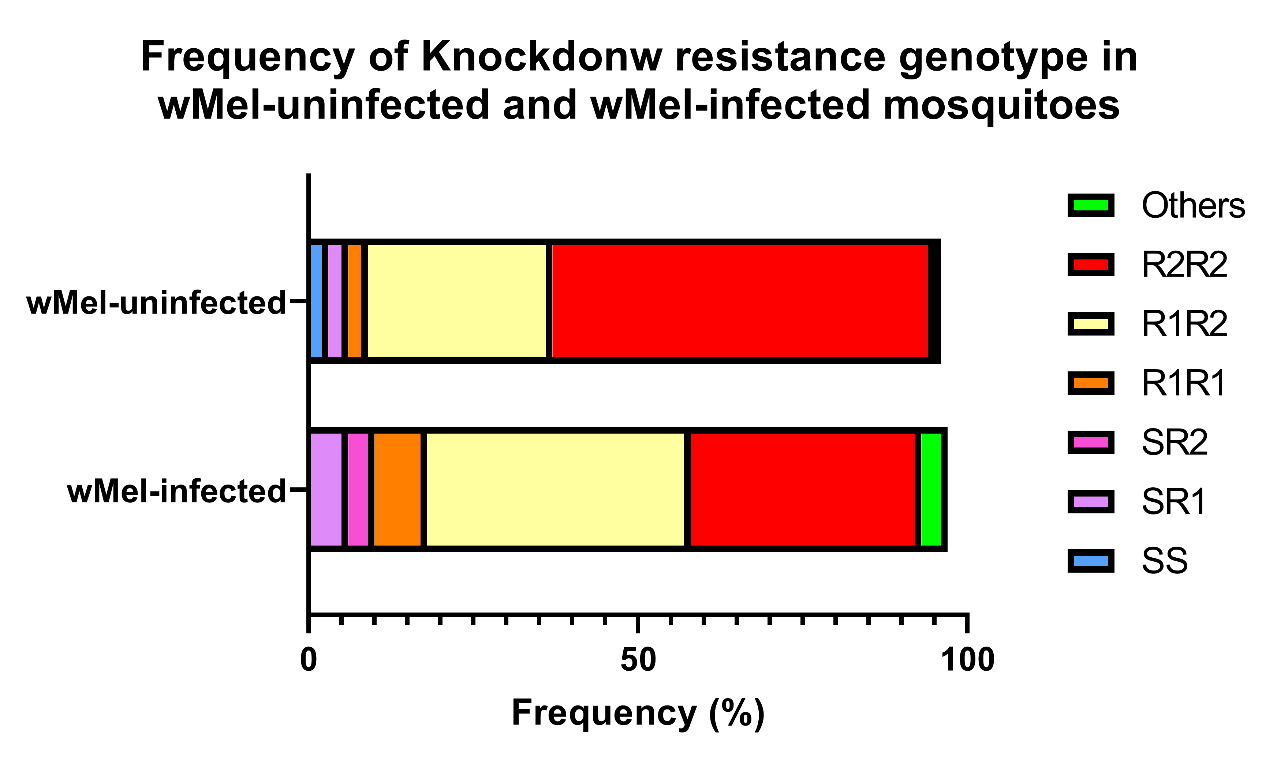

Supplement: S2 Fig — (DOCX) [file pntd.0013372.s006.docx]
